# Long-Term Survival After Venous Thromboembolism: A Prospective Cohort Study

**DOI:** 10.3389/fcvm.2021.749342

**Published:** 2021-10-01

**Authors:** Henning Nilius, Tamara Mertins, Robin Boss, Matthias Knuchel, Eva Blozik, Johanna Anna Kremer Hovinga, Sabine Eichinger, Michael Nagler

**Affiliations:** ^1^University Institute of Clinical Chemistry, Inselspital University Hospital, University of Bern, Bern, Switzerland; ^2^Department of Epidemiology, Maastricht University, Maastricht, Netherlands; ^3^Department of Health Sciences, Helsana Group, Zuerich, Switzerland; ^4^Department of Hematology and Central Hematology Laboratory, Inselspital, Bern University Hospital, and University of Bern, Bern, Switzerland; ^5^Department of Medicine 1, Medical University of Vienna, Vienna, Austria

**Keywords:** cohort study, epidemiology, mortality, thrombosis, venous thrombosis

## Abstract

**Background:** Little is known about long-term survival after the initial treatment of venous thromboembolism (VTE). In a prospective cohort study, we aimed to assess the long-term mortality and key predictor variables relating to disease severity, treatment intensity, and comorbidities.

**Materials and Methods:** Between 1988 and 2018, 6,243 consecutive patients with VTE from a University outpatient unit were prospectively included and followed until December 2019; clinical characteristics, measures of disease severity, and treatment details were recorded. Dates of death were retrieved from the Swiss Central Compensation Office.

**Results:** Overall, 254 deaths occurred over an observation period of 57,212 patient-years. Compared to the Swiss population, the standardized mortality ratio was 1.30 (95% CI: 1.14, 1.47; overall mortality rate: 4.44 per 1,000 patient-years). The following predictors were associated with increased mortality: Unprovoked VTE (hazard ratio [HR]: 5.06; 95% CI: 3.29, 7.77), transient triggering risk factors (HR: 3.46; 95% CI: 2.18, 5.48), previous VTE (HR: 2.05; 95% CI: 1.60, 2.62), pulmonary embolism (HR: 1.45, 95% CI: 1.10, 1.89), permanent anticoagulant treatment (HR: 3.14; 95% CI: 2.40, 4.12), prolonged anticoagulant treatment (7–24 months; HR: 1.70; 95% CI: 1.16, 2.48), and cardiovascular comorbidities. Unprovoked VTE, previous VTE, permanent and prolonged anticoagulation remain independent risk factors after adjustment for age, sex, and comorbidities.

**Conclusion:** Survival after VTE was significantly reduced compared to the Swiss general population, especially in patients with more severe disease, cardiovascular comorbidities, and longer anticoagulant treatment.

## Introduction

Venous thromboembolism (VTE) is a common vascular disorder that contributes significantly to the global disease burden (1). The incidence of VTE is estimated to be 80–270 per 100,000 persons with a short term mortality rate comparable to ischemic stroke (2–4). A significant proportion of patients dies in the acute phase due to heart failure following pulmonary embolism (PE) (5). In patients who survive the acute phase of the disease, the mortality rate is often perceived as low.

To date, knowledge about long-term mortality and its predictors is limited, and previous studies' results are conflicting. Various analyses using data from the Dutch MEGA study (6), the US Olmsted county cohort (7), the Swiss SWITCO65+ cohort (8), and cohorts from Australia (9), and Italy (10, 11) identified malignancy as the most important risk factor for mortality. Advanced age, male sex, and cardiovascular comorbidities were reported as major risk factors in an analysis of the Taiwanese National Health Insurance database (12), the SWITCO65+ cohort (8), and the Olmsted county cohort (7). In contrast, unprovoked VTE was a major risk factor for mortality in the TEHS cohort (13), and in a Norwegian cohort (14). Of note, long-term heparin treatment was observed as a risk factor in the Italian MASTER registry (11). Interpretation of previous studies is, however, difficult because of various methodological limitations. Either, a retrospective or case-control design was used, a small range of variables reflecting disease severity, treatment intensity, and comorbidities was recorded, the sample size was small, or the observation time was short. The question arises, whether the long-term survival of VTE patients is indeed low, and which factors contribute to mortality. Do comorbidities solely determine the mortality, or do disease severity and treatment intensity play a role?

Aiming to comprehensively assess the effect of disease severity, treatment intensity, and comorbidities on the long-term survival of patients with VTE, we conducted a large, prospective cohort study with patient follow-up for up to 30 years and recorded a broad set of predictor variables.

## Materials and Methods

### Study Design, Setting, and Population

**Figure 1:**
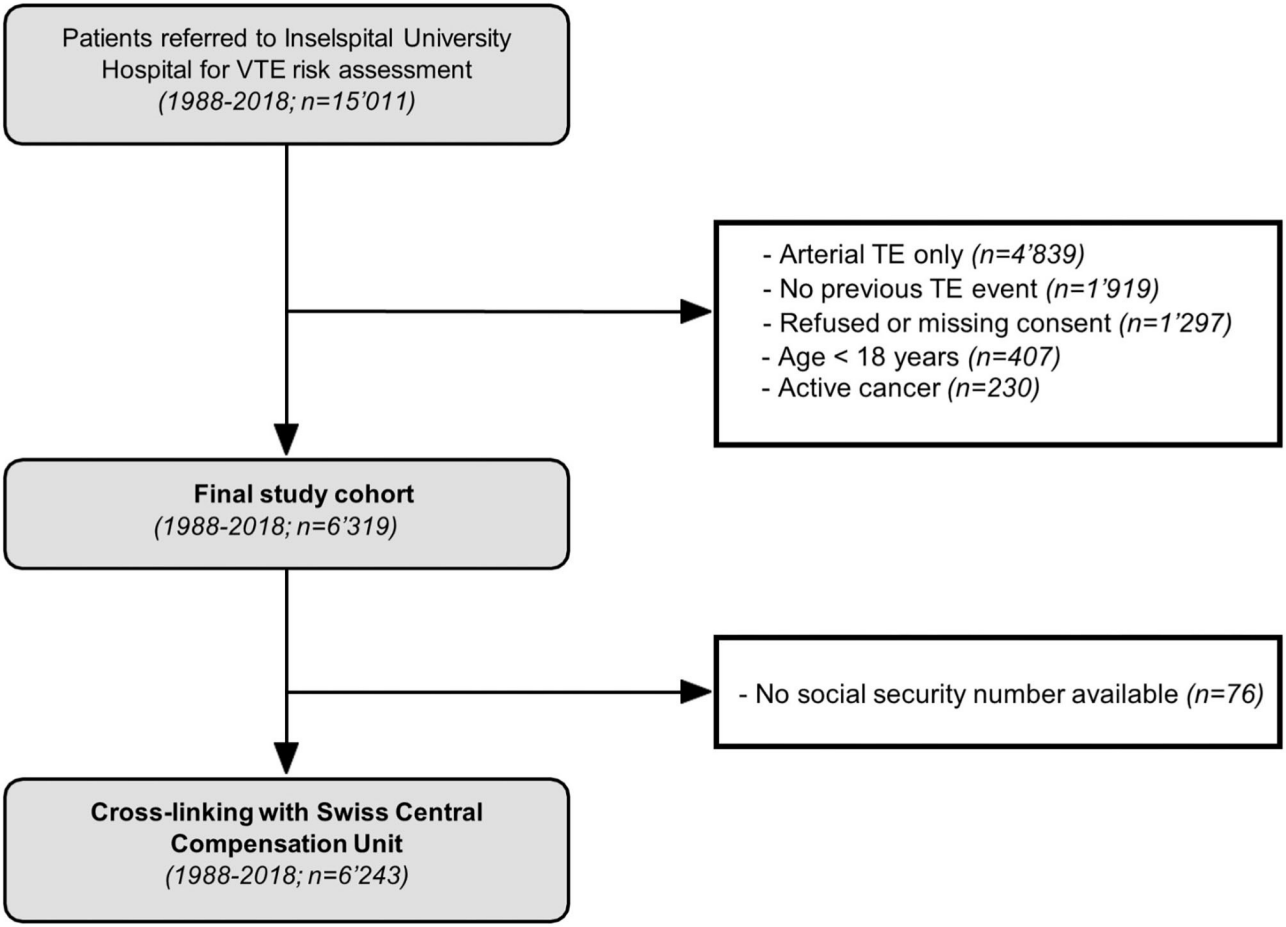
Flow of patients.

SeProV (“Secondary prophylaxis of venous thromboembolism in the Greater Bern Area”) is a long-term prospective cohort study conducted in Bern, Switzerland. Between 1988 and 2018, all consecutive patients, which were referred for VTE risk assessment to a specialized outpatient unit at the Inselspital, Bern University Hospital, were included and followed until December 2019. Patients were usually referred following (a) venous thromboembolism, (b) arterial thromboembolism, (c) or positive family history (Figure 1). We expect that the majority of patients with VTE have been referred to our center. Out of this cohort, the following inclusion criteria were applied: (1) objectively confirmed VTE [venography or duplex sonography in case of deep vein thrombosis (DVT), spiral computed tomography, ventilation-perfusion scan or pulmonary angiography in case of PE, and various techniques in case of rare VTE], (2) referral for VTE risk assessment, and (3) age above 18 years. Exclusion criteria were (a) refused informed consent, and (b) active cancer.

The Inselspital is one of the largest academic hospitals in Switzerland and has a catchment population of ~2 million inhabitants. A formal risk assessment is recommended in all patients considered for secondary prophylaxis of VTE and the specialized outpatient unit of the Inselspital is the most important center for risk assessment in the greater Bern area. We, therefore, expect that the majority of patients with VTE have been referred to our center. This study was approved by the ethical committee (Kantonale Ethikkommission Bern; #18-00389) and informed consent was obtained from all participants. The study was conducted in accordance with the Declaration of Helsinki.

### Data Collection

Patients were screened and included during VTE risk assessment in a specialized outpatient unit. In our setting, VTE risk assessment is recommended after completion of initial anticoagulant treatment following VTE. Clinical characteristics were prospectively recorded in a structured assessment form by the responsible resident and then reviewed by the attending physician. The consistency of data was additionally checked by a trained study nurse and a trained investigator. Parameters of interest and corresponding values were defined beforehand, and the definitions were included in the data collection forms. The following data were obtained: age, sex, type of index VTE, presence of triggering risk factors, presence of previous VTE, family history of VTE, presence of arterial thrombosis, smoking status, obesity, presence of systemic diseases including inflammatory bowel disease and systemic lupus erythematodes, diabetes, coronary heart disease without myocardial infarction, chronic lung disease, hypertension, active cancer, chronic kidney disease, and anemia. Treatment details including duration of anticoagulation were additionally recorded. By matching the patients with the Unique Person Identification registry of the Central Compensation Office (ZAS) which provides complete death data of all Swiss residents, the vital status, and time point of death were acquired. All Swiss civil registries automatically report to this database, and it is regarded as complete. The matching was based on a national social insurance number (SV number) of Swiss residents, which is unique, anonymous, and not based on personal characteristics. Patients that were not found in the registry, e.g., due to migration to another country, were censored at the time of the last visit at the Inselspital Bern.

### Definition of Variables

Death was defined as a respective notification in the Unique Person Identification registry of the Central Compensation Office (ZAS). Categorization of the index events and the triggering risk factors was done according to current guidelines and verified again in 2018 using original hospital records. As indicators of disease severity, the type of index VTE, presence of triggering risk factors, presence of previous VTE, and family history were considered. Mutual exclusive groups were created with regard to the type of index event. All patients with PE were categorized as “Pulmonary embolism,” regardless of concomitant thromboembolic events. Patients with DVT (lower leg, upper leg, and pelvic vein thrombosis) without PE were categorized as “Deep vein thrombosis,” regardless of additional thromboembolic events. Patients with all other VTE, but not PE or DVT were classified as “other VTE” (cerebral vein thrombosis, mesenteric thrombosis, portal vein thrombosis, upper extremity vein thrombosis, superficial vein thrombosis, and muscle vein thrombosis). Unprovoked VTE was defined as VTE in the absence of a reversible risk factor (surgery, contraceptive use, pregnancy, long-distance air or car travel of more than 10 h, immobilization, and traumatic injury at most 3 months before the VTE) (15, 16). Surgery, immobilization, long-distance travel of more than 10 h, and traumatic injury within 3 months before the VTE were defined as transient risk factors. Active cancer was defined as diagnosed within the previous 6 months, recurrent, regionally advanced or metastatic cancer, cancer for which treatment had been administered within 6 months, or hematological cancer that is not in complete remission (17). Pregnancy and anti-contraceptive use were classified as hormone-related risk factors. As an indicator of treatment intensity, the duration of anticoagulant treatment was categorized as follows: (1) ≤6 months, (2) 7–24 months (prolonged), (3) >24 months (permanent) (18). The smoking status was recorded as either active smoker or non-smoker. Patients with a BMI > 30 kg/m^2^ were categorized as obese. The following comorbidities were recorded as present or absent: arterial thrombosis, hypertension, diabetes mellitus, stroke, coronary artery disease, kidney failure, pulmonary disease, kidney disease, systemic diseases, and anemia.

### Statistical Analysis

The baseline characteristics of the predictor variables were presented by sex. The variables were described as numbers (percentages) and median (interquartile range) as appropriate,

A maximum of 1.6% of the data was missing for each of the recorded covariates and was deemed either missing at random or missing completely at random. Therefore, we decided to impute the missing values using an algorithm based on a random forest approach implemented in the “missForest” package of “R.” (19).

We calculated the mortality rates per 1,000 patient-years (py) for the whole cohort and each predictor. The accumulated person-time at risk and the number of deaths per subgroup were calculated. Sex and age-specific standardized mortality ratios (SMR) were calculated by indirect standardization to compare the mortality in the SeProV cohort with the general Swiss population. The sex and age-specific mortality rates per 100,000 persons of the Swiss population were obtained from the Swiss Federal Statistical Office (latest available rates from 2017). Ninety-five percent confidence intervals were calculated using the method described by Vandenbroucke (20). The survival curve for the whole cohort was calculated using a Kaplan–Meier estimate. The Kaplan–Meier curves of groups with and without risk factors were compared and a log-rank test was employed to test the difference in cumulative hazard between the groups. A univariate Cox proportional-hazards model was used for each parameter. Hazard ratios (HR) and their corresponding 95% confidence intervals were reported. Multivariate models were created by separately entering one of the predictors of disease severity and treatment intensity (index event, previous VTE, family history of VTE, triggering risk factors, and duration of anticoagulant treatment) adjusting for age, sex, and comorbidities (arterial thrombosis, obesity, smoking status, systemic diseases, diabetes mellitus, coronary artery disease, pulmonary disease, hypertension, kidney disease, stroke, and anemia). Hazard Ratios and their confidence intervals were calculated from the coefficients. The HRs and their respective 95% confidence intervals for age, sex, smoking status, and comorbidities were obtained from a model including all comorbidities but none of the predictors of disease severity and treatment intensity.

For both the Kaplan–Meier and the Cox-Proportional-Hazard model the assumption of non-informative censoring was tested by comparing an optimistic model, in which all censored times are seen as longest possible survival, to a pessimistic model, in which all censored times are seen as deaths and comparing them. If the results pointed in different directions, we performed a sensitivity analysis and investigated the reasons for informative censoring. A histogram was created plotting the date of assessment on the *x*-axis and the number of patients per vital status (alive, dead, and censored) on the *y*-axis. To test the assumption of constant hazard the scaled Schoenfeld residuals were calculated for each predictor and the correlations with time were tested, both with a statistical test as well as visually.

Two sensitivity analyses were performed for different periods of patient enrolment and to account for potential effects of a wide range of “other VTE.” Multivariate Cox-Proportional-Hazard models for patients enrolled between 1988–1997, 1998–2007, and 2008–2018 were fitted. Another analysis was done considering patients with DVT and PE only. All analyses were performed using the statistical software environment “R” (21).

## Results

### Study Population and Patient Characteristics

Among 15,011 patients referred between 1988 and 2018 to the Inselspital for thromboembolic risk assessment, 6,243 patients with objectively confirmed VTE were included in the final study cohort after a mean 4.2 months of anticoagulation (Figure 1). The median observation time was 8.13 years, adding up to a total of 57,212 py. The median time between index event and VTE risk assessment (inclusion into the study) was 1.2 years (interquartile range [IQR]: 0.78, 1.57). The median age was 44.60 years (IQR: 32.55, 49.71) and 58.7% of the patients were female. Pulmonary embolism was the index event in 43.1% of the patients (*n* = 2,679), and DVT in 40.3% (*n* = 2,506). Unprovoked VTE was present in 42.2% (*n* = 2,622) and 31.2% (*n* = 1,946) had previous VTE. Permanent anticoagulant treatment was given to 28.6% of the patients (*n* = 1,786). Detailed patient characteristics are reported in Table 1.

**Table 1:** Characteristics of 6,243 patients with venous thromboembolism followed for a median of 8.13 years.

	**Overall**	**Female**	**Male**	**Missing values**
*n*	6,243	3,663	2,580	
Median age (IQR)—y	44.60 (32.55, 56.09)	39.17 (29.36, 52.73)	49.47 (40.24, 59.28)	
**Index event—no. (%)**				30 (0.5)
Deep vein thrombosis	2,506 (40.3)	1,526 (41.9)	980 (38.1)	
Pulmonary embolism	2,679 (43.1)	1,438 (39.5)	1,241 (48.3)	
Other VTE	1,028 (16.5)	680 (18.7)	348 (13.5)	
Previous VTE—no. (%)	1,946 (31.2)	952 (27.2)	994 (36.4)	15 (0.2)
Family history—no. (%)	1,968 (31.6)	1,180 (32.4)	788 (30.6)	24 (0.4)
**Triggering risk factors—no. (%)**				30 (0.5)
Pregnancy	324 (5.2)	324 (8.8)	0 (0)	
Estrogen use	1,570 (25.3)	1,570 (42.8)	0 (0)	
Transient risk factors	1,697 (27.3)	764 (20.9)	933 (36.4)	
Unprovoked VTE	2,622 (42.2)	992 (27.2)	1,630 (63.5)	
**Duration of anticoagulant treatment—no. (%)**				7 (0.1)
<6 months	3,617 (58.0)	2,362 (64.5)	1,255 (48.7)	
Prolonged	833 (13.4)	522 (14.3)	311 (12.1)	
Permanent	1,786 (28.6)	777 (21.2)	1,009 (39.2)	
Arterial thrombosis—no. (%)	285 (4.6)	124 (3.4)	161 (6.2)	0 (0.0)
Smoking—no. (%)	1,445 (23.5)	844 (23.3)	601 (23.7)	87 (1.4)
Obesity—no. (%)	1,792 (29.2)	995 (27.7)	797 (31.3)	99 (1.6)
Systemic disease—no. (%)	376 (6.0)	217 (5.9)	159 (6.2)	16 (0.3)
Diabetes mellitus—no. (%)	171 (2.7)	79 (2.2)	92 (3.6)	18 (0.3)
Coronary artery disease—no. (%)	259 (4.2)	89 (2.4)	170 (6.6)	18 (0.3)
Pulmonary disease—no. (%)	251 (4.0)	141 (3.9)	110 (4.3)	17 (0.3)
Hypertension—no. (%)	803 (12.9)	373 (10.2)	430 (16.7)	18 (0.3)
Kidney disease—no. (%)	139 (2.2)	56 (1.5)	83 (3.2)	16 (0.3)
Stroke—no. (%)	191 (3.1)	89 (2.4)	102 (4.0)	17 (0.3)
Anemia—no. (%)	217 (3.5)	160 (4.4)	57 (2.2)	17 (0.3)

*Baseline demographics, measures of disease severity, treatment intensity, and comorbidities are reported by sex. IQR, interquartile range; no., number of patients; VTE, venous thromboembolism*.

### Mortality

**Figure 2:**
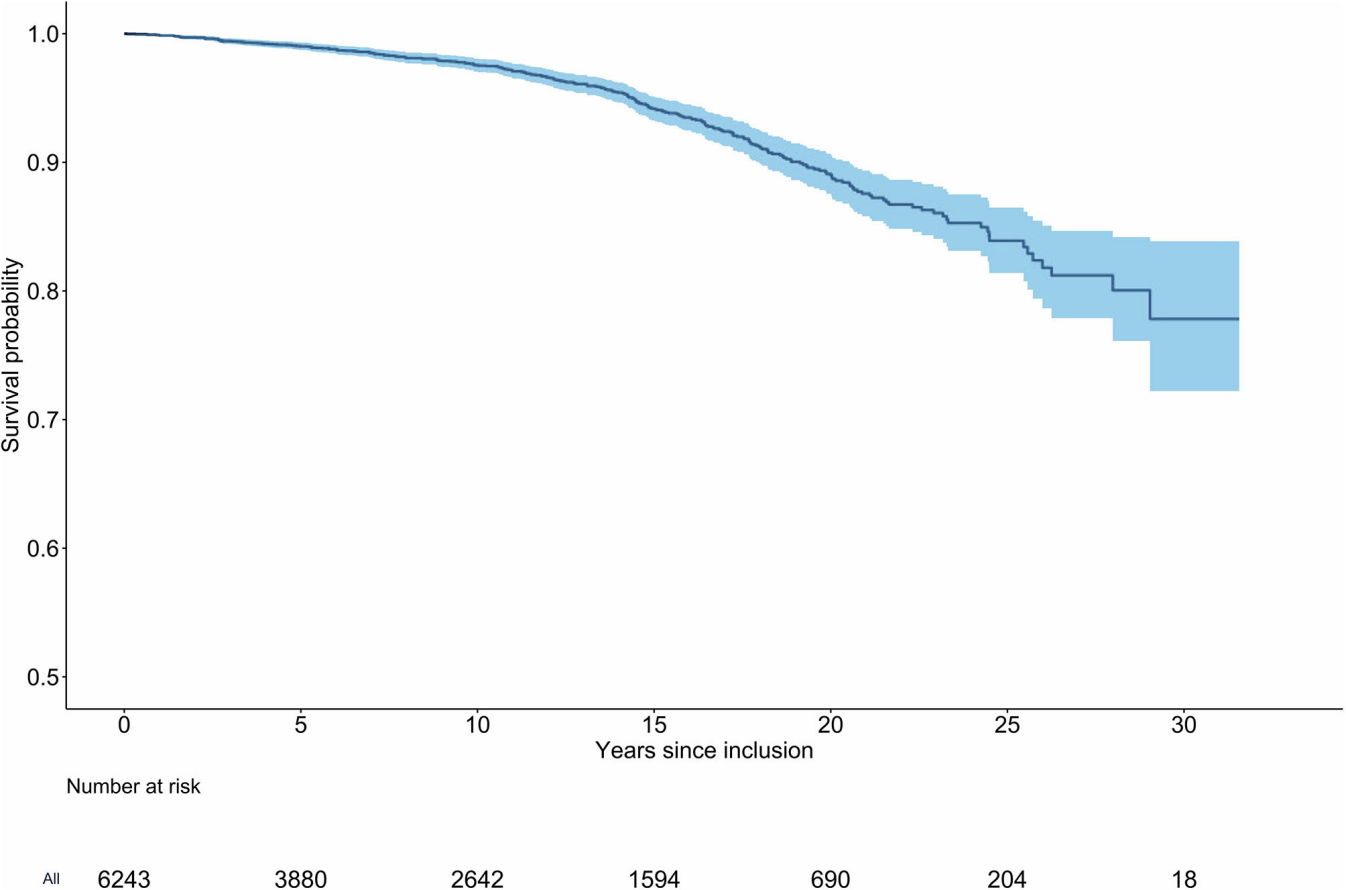
Kaplan-Meier curve of survival for patients with venous thromboembolism. Two-hundred fifty-four deaths were observed in a cumulative observation period of 57′212 patient-years (6′243 patients). The median time between index event and inclusion into the study was 1.2 years (IQR: 0.78, 1.85).

Two-hundred fifty-four patients died amounting to a mortality rate of 4.44 per 1,000 py (95% confidence interval [CI]: 3.89, 4.99). Detailed results in various subgroups are given in Table 2. In a Kaplan Meier analysis the cumulative probability of survival was 99.0% (95% CI: 98.7, 99.3%) after 5 years, 97.6% (95% CI: 97.0, 98.1%) after 10 years, 94.2% (95% CI: 93.3, 95.1%) after 15 years, and 89.1% (95% CI: 87.6, 90.7%) after 20 years (Figure 2). Standardized to the Swiss general population, the SMR was 1.30 (95% CI: 1.14, 1.47). In female patients, the mortality rate was 3.33 per 1,000 py (95% CI: 2.72, 3.94; SMR: 1.48, 95% CI: 1.22, 1.77). In male patients, the mortality rate was 5.98 per 1,000 py (95% CI: 4.99, 6.97; SMR: 1.18, 95% CI: 0.99, 1.39). The mortality rate in patients with previous VTE was 6.96 per 1,000 py (95% CI: 5.78, 8.13) and the SMR was 1.56 (95% CI: 1.30, 1.84). In patients with unprovoked VTE, the mortality rate was 6.51 per 1,000 py (95% CI: 5.49, 7.54; SMR: 1.32, 95% CI: 1.11, 1.54) and in patients with pulmonary embolism as index event, the mortality rate was 5.29 per 1,000 py (95% CI: 4.37, 6.20; SMR: 1.31, 95% CI: 1.09, 1.55). Patients with permanent anticoagulant treatment had a mortality rate of 7.80 per 1,000 py (95% CI: 6.41, 9.20) and an SMR of 1.44 (95% CI: 1.19, 1.71). With regard to comorbidities, patients with coronary artery disease had a mortality rate of 13.3 per 1,000 py (95% CI: 8.45, 18.1; SMR: 1.48, 95% CI: 0.98, 2.08) and patients with diabetes mellitus had a rate of 14.6 per 1,000 py (95% CI: 8.35, 20.8; SMR: 2.62, 95% CI: 1.60, 3.89).

**Table 2:** Long-term mortality by risk factors.

	**Deaths**	**Observation period (py)**	**Mortality rate per 1,000 py**	**Standardized mortality ratio**
Overall	254	57,212	4.44 (3.89, 4.99)	1.30 (1.14, 1.47)
Females	114	34,274	3.33 (2.72, 3.94)	1.48 (1.22, 1.77)
Males	140	23,411	5.98(4.99, 6.97)	1.18 (0.99, 1.39)
**Index event**				
Deep vein thrombosis	91	23,908	3.81 (3.02, 4.59)	1.29 (1.04, 1.58)
Pulmonary embolism	128	24,208	5.29 (4.37, 6.20)	1.31 (1.09, 1.55)
Other VTE	35	9,569	3.66 (2.45, 4.87)	1.28 (0.88, 1.75)
Previous VTE	135	19,407	6.96 (5.78, 8.13)	1.56 (1.30, 1.84)
Family history	67	17,281	3.88 (2.95, 4.81)	1.14 (0.88, 1.44)
**Triggering risk factors**				
Pregnancy and estrogen use	24	17,756	1.35 (0.81, 1.89)	1.27 (0.80, 1.84)
Transient risk factors	75	16,129	4.65 (3.60, 5.70)	1.27 (0.99, 1.58)
Unprovoked VTE	155	23,801	6.51 (5.49, 7.54)	1.32 (1.11, 1.54)
**Duration of anticoagulant treatment**				
≤ 6 months	97	34,288	2.83 (2.27, 3.39)	1.11 (0.90, 1.35)
Prolonged	37	8,020	4.61 (3.13, 6.10)	1.49 (1.04, 2.02)
Permanent	120	15,377	7.80 (6.41, 9.20)	1.44 (1.19, 1.71)
Arterial thrombosis	19	2,483	7.65 (4.21, 11.1)	1.30 (0.77, 1.96)
Smoking	69	14,715	4.69 (3.58, 5.80)	2.57 (1.99, 3.23)
Obesity	94	16,985	5.53 (4.42, 6.65)	1.57 (1.27, 1.92)
Systemic disease	25	4,003	6.25 (3.80, 8.69)	2.05 (1.31, 2.95)
Diabetes mellitus	21	1,440	14.6 (8.35, 20.8)	2.62 (1.60, 3.89)
Coronary artery disease	29	2,183	13.3 (8.45, 18.1)	1.48 (0.98, 2.08)
Pulmonary disease	18	2,380	7.56 (4.07, 11.1)	1.53 (0.89, 2.33)
Hypertension	74	7,363	10.1 (7.76, 12.3)	1.46 (1.14, 1.82)
Kidney disease	25	1,224	20.4 (12.4, 28.4)	2.14 (1.37, 3.08)
Stroke	17	1,671	10.2 (5.34, 15.0)	1.56 (0.89, 2.40)
Anemia	18	1,959	9.19 (4.94, 13.4)	2.57 (1.50, 3.93)

*Mortality rates and standardized mortality ratios are reported (Reference: Swiss general population). VTE, venous thromboembolism; py, patient-years*.

### Risk Factors for Mortality

Mortality was associated with various demographic characteristics, measures of disease severity, treatment intensity, as well as comorbidities. In univariate analysis, the HR of male sex was 1.87 (95% CI: 1.46, 2.40). Regarding disease severity, the hazard ratio of previous VTE was 2.05 (95% CI: 1.60, 2.62). The HR of PE as an index event was 1.45 (95% CI: 1.10, 1.89), and the HR of unprovoked VTE was 5.06 (95% CI: 3.29, 7.77). Considering treatment intensity, the HR of prolonged anticoagulant treatment (7–24 months) was 1.70 (95% CI: 1.16, 2.48), and the HR of permanent anticoagulant treatment was 3.14 (95% CI: 2.40, 4.12). Various cardiovascular comorbidities were associated with an increased mortality in univariate analysis, details are reported in Table 3 and Kaplan–Meier-Curve for selected covariates in Supplementary Figure 1.

**Table 3:** Predictors of long-term mortality: Baseline demographics, measures of disease severity, treatment intensity, and comorbidities.

	**Univariate**		Multivariate^*^	
	**HR**	**95% CI**	**HR**	**95% CI**
Age	1.09	1.08, 1.10	1.09	1.08, 1.11
Male Sex	1.87	1.46, 2.40	1.29	1.00, 1.65
**Index event**				
Deep vein thrombosis	–	–	–	–
Pulmonary embolism	1.45	1.10, 1.89	1.18	0.90, 1.56
Other VTE	0.99	0.67, 1.46	0.91	0.62, 1.35
Previous VTE	2.05	1.60, 2.62	1.33	1.04, 1.71
Family history	0.86	0.65, 1.13	0.87	0.66, 1.16
**Triggering risk factors**				
Pregnancy and estrogen use	–	–	–	–
Transient risk factors	3.46	2.18, 5.48	1.63	0.99, 2.66
Unprovoked VTE	5.06	3.29, 7.77	1.69	1.05, 2.72
**Duration of anticoagulant treatment**				
≤ 6 months	–	–	–	–
Prolonged	1.70	1.16, 2.48	1.53	1.04, 2.24
Permanent	3.14	2.40, 4.12	1.66	1.25, 2.21
Arterial thrombosis	1.89	1.19, 3.02	0.76	0.39, 1.46
Smoking	0.98	0.75, 1.30	1.91	1.42, 2.57
Obesity	1.40	1.09, 1.81	1.15	0.88, 1.50
Systemic disease	1.32	0.88, 2.00	1.07	0.70, 1.63
Diabetes mellitus	4.01	2.56, 6.29	2.09	1.31, 3.32
Coronary artery disease	3.92	2.66, 5.78	1.56	1.03, 2.37
Pulmonary disease	1.88	1.16, 3.03	1.38	0.85, 2.24
Hypertension	3.01	2.29, 3.94	1.19	0.89, 1.59
Kidney disease	5.58	3.69, 8.44	2.31	1.47, 3.63
Stroke	2.51	1.53. 4.11	1.38	0.69, 2.76
Anemia	2.23	1.38, 3.60	2.34	1.42, 3.87

*VTE, venous thromboembolism; HR, hazard ratio; CI, confidence interval*.

* *Adjusted by age, sex, and comorbidities*.

Adjusted for age, sex, and all comorbidities listed in Table 3, HR previous VTE (adjusted HR: 1.33, 95% CI: 1.04, 1.71), unprovoked VTE (adjusted HR: 1.69, 95% CI: 1.05, 2.72), prolonged anticoagulant treatment (adjusted HR: 1.53, 95% CI: 1.04, 2.25), and permanent anticoagulant treatment (adjusted HR: 1.66, 95% CI: 1.25, 2.21) were independent risk factors for mortality.

The assumption of non-informative censoring was violated as the pessimistic model for sex was not statistically significant (HR: 0.94, 95% CI: 0.86, 1.02), whereas the optimistic model was significant (HR: 1.93, 95% CI: 1.51, 2.47) (Supplementary Table 1). Considering the histogram, the relative number of censored patients remained the same until 2008, and from 2008 on decreased (Supplementary Figure 2). The sensitivity analysis, however, showed no significant difference when excluding the censored patients (Supplementary Table 2). The assumption of constant hazard, however, holds for all models created (Supplementary Table 3; Supplementary Figure 3). No significant differences were identified in the two sensitivity analyses considering different periods of enrolment and patients with DVT and PE only (Supplementary Tables 4, 5). However, the models fitted to different enrolment times became unstable due to small sample sizes (particularly with regard to the time period 1988 to 1997).

## Discussion

We conducted a large, prospective cohort study, recording a broad set of predictor variables, and following for up to 30 years, to comprehensively assess the effect of disease severity, treatment intensity, and comorbidities on the long-term survival of patients with VTE. Two-hundred and fifty-four deaths were observed among 6,243 patients over an observation period of 57,212 patient-years. Compared to the general Swiss population, long-term mortality of VTE patients was increased by 30%. Besides higher age and male sex, increased mortality was associated with measures of disease severity, treatment intensity, and cardiovascular comorbidities. Previous VTE, unprovoked VTE, prolonged, and permanent anticoagulant treatment were independent risk factors for mortality.

Even though a less strict study design was used in most publications, some aspects were observed previously. Increased mortality was found by two large case-control studies and one retrospective cohort study (6, 12, 22), but not in a smaller study conducted in young women comparing the mortality rate with the general population (23). Older age and male sex were identified as a risk factor in the SWITCO65+ cohort (8), in the Olmsted county cohort (7), and in the Taiwanese National Health Insurance study (12). Diabetes mellitus and other cardiovascular risk factors and comorbidities were reported in an analysis of the Taiwanese National Health Insurance database (12), the SWITCO65+ cohort (8), and the Olmsted county cohort (7). As a measure of disease severity, unprovoked VTE was observed as a risk factor in the TEHS cohort (13), and in a Norwegian cohort (14). As additional disease severity measures, we found PE as index event, previous VTE, and transient triggering factors to be risk factors for mortality. One previous study analyzed long-term heparin-treatment as a measure of treatment intensity (11), which was associated with mortality. In our study, prolonged and permanent anticoagulant treatment were associated with increased mortality (HR: 1.53 and 1.66, respectively). To explore causal relations, we also adjusted disease severity measures and treatment intensity for age, sex, and cardiovascular comorbidities, leaving prolonged and permanent anticoagulant treatment as an independent predictor of mortality.

Our study has several strengths. We included a large number of patients and followed them prospectively for up to 30 years. We recorded a broad set of predictor variables reflecting measures of disease severity, treatment intensity, and comorbidities. The quality of the data is high, and the percentage of missing values is low.

As with every study, methodological limitations and potential sources of bias appear. First, selection bias is a potential source of bias in this study. A heterogeneous group of general practitioners and specialists referred the patients, and unconscious influences may have affected referral decisions. In our cohort, the patients were relatively young (mean 44.6 years) and the proportion of individuals with pregnancy or estrogen-associated VTE was relatively high (30.5%). Thus, the results might differ in an older population, which must be clarified in future studies. Secondly, data on whether the general practitioner prescribed vitamin K antagonists or direct oral anticoagulants (DOAC) following the risk assessment are unavailable. This selection might have affected the results since DOAC were increasingly used since 2012. Even though a sensitivity analysis by time period did not find relevant differences, we cannot fully exclude that this might have influenced the results. Thirdly, clinical data were collected during the routine assessment by a trained physician using a standardized questionnaire. However, some questions, e.g., smoking habits, rely on self-reported answers, and are, therefore, prone to information bias. Fourthly, clinical data collection was continued until 2018 and the time points of death were obtained in 2019. Therefore, patients who were assessed early in the study have a much longer follow-up time than patients who were assessed more recently. Therefore, there might be an underestimation of associations of predictors that are more common now than 20 years ago. Fifthly, the assumption of uninformative censoring did not hold. The most likely explanation for this is the change of the SV number in Switzerland in 2008. However, we assume that the censoring before 2008 is random and the sensitivity analysis did not show a significant change in the effect of the predictors.

Our results confirm previous data that the long-term survival of patients with VTE is reduced. Patients with more severe disease and more intense treatment are at higher risk of death. This effect can be partially explained by cardiovascular comorbidities, suggesting an association between both clinical situations. However, more severe disease and more intense treatment remain independent risk factors for mortality. Close monitoring and reduced-intensity treatment schemes are potential targets to improve long-term care in patients with severe VTE. Reduced dosages of direct oral anticoagulants might fill this gap. Future studies shall verify our results in different settings and investigate the potential benefits of reduced-intensity treatment schemes in patients that require permanent anticoagulant treatment.

In a large, prospective cohort study, recording a broad set of predictor variables, and following patients for up to 30 years, we comprehensively assessed the effect of disease severity, treatment intensity, and comorbidities on the long-term survival of patients with VTE. Our data indicate that the long-term survival of patients with VTE is indeed restricted. Survival after VTE was significantly reduced in patients with more severe disease, cardiovascular comorbidities, and longer anticoagulant treatment. Our results need to be verified in other settings and populations, especially older patients.

## Data Availability Statement

The raw data supporting the conclusions of this article will be made available by the authors, without undue reservation.

## Ethics Statement

The studies involving human participants were reviewed and approved by Ethikkommission Kanton Bern, 3010 Bern, Switzerland. The patients/participants provided their written informed consent to participate in this study.

## Author Contributions

HN analyzed the data, contributed to interpretation, and wrote the manuscript. TM, RB, and MK collected data and contributed to interpretation and manuscript. EB, JK, and SE contributed to study design, data analysis, interpretation, and manuscript. MN designed the study, analyzed the data, interpreted the findings, and wrote the manuscript. All authors contributed to the article and approved the submitted version.

## Funding

This investigator-initiated study was supported by an unrestricted research grant of Bayer Healthcare. MN is supported by a research grant of the Swiss National Science Foundation (#179334).

## Conflict of Interest

This study received funding from an unrestricted research grant of Bayer Healthcare. The funder was not involved in the study design, collection, analysis, interpretation of data, the writing of this article or the decision to submit it for publication. EB reports grants from Novartis, grants from MSD, grants from Vifor, grants from Bayer, grants from Swiss Cancer Research Foundation, outside the submitted work. JK reports grants from Baxalta US Inc., member of the Takeda group of companies, personal fees from Shire, member of the Takeda group of companies, personal fees from Ablynx, now part of Sanofi, personal fees from Roche, from SOBI, from Federal Office of Public Health, Switzerland, outside the submitted work; and The Hemophilia Comprehensive Care Center (HCCC) is part of the Department of Hematology and Central Hematology Laboratory, Inselspital, Bern University Hospital, which receives third party funds for the project “Interprofessional Hemophilia Care” by Bayer, CSL-Behring, Octapharma, Novo Nordisk, Roche, and Sobi. All fees or honoraria go to JK's institution (Insel Gruppe AG, Inselspital, Bern University Hospital). SE reports personal fees from Bayer, personal fees from Pfizer, personal fees from Daiichi-Sankyo, personal fees from Boehringer-Ingelheim, personal fees from BMS, personal fees from CSL-Behring, outside the submitted work. The remaining authors declare that the research was conducted in the absence of any commercial or financial relationships that could be construed as a potential conflict of interest.

## Publisher's Note

All claims expressed in this article are solely those of the authors and do not necessarily represent those of their affiliated organizations, or those of the publisher, the editors and the reviewers. Any product that may be evaluated in this article, or claim that may be made by its manufacturer, is not guaranteed or endorsed by the publisher.

## References

[1] Raskob GE Angchaisuksiri P Blanco AN Buller H Gallus A Hunt BJ. Thrombosis: a major contributor to global disease burden: ISTH steering committee for world thrombosis day the members of the ISTH steering committee for World Thrombosis Day. Thromb Res. (2014) 134:931–8. 10.1016/j.thromres.2014.08.014 25312343

[2] Woo KS Tse LKK Tse CY Metreweli C Vallance-Owen J. The prevalence and pattern of pulmonary thromboembolism in the Chinese in Hong Kong. Int J Cardiol. (1988) 20:373–80. 10.1016/0167-5273(88)90291-4 3262592

[3] Holst AG Jensen G Prescott E. Risk factors for venous thromboembolism: results from the Copenhagen City Heart Study. Circulation. (2010) 121:1896–903. 10.1161/CIRCULATIONAHA.109.921460 20404252

[4] Wendelboe AM Raskob GE. Global burden of thrombosis: epidemiologic aspects. Circ Res. (2016) 118:1340–7. 10.1161/CIRCRESAHA.115.306841 27126645

[5] Laporte S Mismetti P Décousus H Uresandi F Otero R Lobo JL. Clinical predictors for fatal pulmonary embolism in 15 520 patients with venous thromboembolism: findings from the Registro Informatizado de la Enfermedad TromboEmbolica venosa (RIETE) registry. Circulation. (2008) 117:1711–16. 10.1161/CIRCULATIONAHA.107.726232 18347212

[6] Flinterman LE van Hylckama Vlieg A Cannegieter SC Rosendaal FR. Long-term survival in a large cohort of patients with venous thrombosis: incidence and predictors. PLoS Med. (2012) 9:e1001155. 10.1371/journal.pmed.1001155 22253578 PMC3254666

[7] Heit JA Silverstein MD Mohr DN Petterson TM O'Fallon WM Melton LJ. Predictors of survival after deep vein thrombosis and pulmonary embolism: a population-based, cohort study. Arch Intern Med. (1999) 159:445–53. 10.1001/archinte.159.5.445 10074952

[8] Faller N Limacher A Méan M Righini M Aschwanden M Beer JH. Predictors and causes of long-term mortality in elderly patients with acute venous thromboembolism: a prospective cohort study. Am J Med. (2017) 130:198–206. 10.1016/j.amjmed.2016.09.008 27742261

[9] Chwan Ng AC Chung T Yong ASC Wong HSP Celermajer DS Kritharides L. Long-term cardiovascular and noncardiovascular mortality of 1023 patients with confirmed acute pulmonary embolism. Circ Cardiovasc Qual Outcomes. (2011) 4:122–8. 10.1161/CIRCOUTCOMES.110.958397 21098781

[10] Prandoni P Villalta S Bagatella P Rossi L Marchiori A Piccioli A. The clinical course of deep-vein thrombosis. Prospective long-term follow-up of 528 symptomatic patients. Haematologica. (1997) 82:423–8. 9299855

[11] Verso M Agnelli G Ageno W Imberti D Moia M Palareti G. Long-term death and recurrence in patients with acute venous thromboembolism: the MASTER registry. Thromb Res. (2012) 130:369–73. 10.1016/j.thromres.2012.04.003 22583838

[12] Chang WT Chang CL Ho CH Hong CS Wang JJ Chen ZC. Long-term effects of unprovoked venous thromboembolism on mortality and major cardiovascular events. J Am Heart Assoc. (2017) 6:e005466. 10.1161/JAHA.117.005466 28468786 PMC5524092

[13] Ljungqvist M Holmström M Kieler H Odeberg J Lärfars G. Cardiovascular disease and mortality after a first episode of venous thromboembolism in young and middle-aged women. Thromb Res. (2016) 138:80–5. 10.1016/j.thromres.2015.11.039 26826509

[14] Andresen MS Sandven I Brunborg C Njaastad AM Strekerud F Abdelnoor M. Mortality and recurrence after treatment of VTE: Long term follow-up of patients with good life-expectancy. Thromb Res. (2011) 127:540–6. 10.1016/j.thromres.2011.02.017 21435698

[15] Kearon C Ageno W Cannegieter SC Cosmi B Geersing GJ Kyrle PA. Categorization of patients as having provoked or unprovoked venous thromboembolism: guidance from the SSC of ISTH. J Thromb Haemost. (2016) 14:1480–3. 10.1111/jth.13336 27428935

[16] Nagler M Angelillo-Scherrer A Méan M Limacher A Abbal C Righini M. Long-term outcomes of elderly patients with CYP2C9 and VKORC1 variants treated with vitamin K antagonists. J Thromb Haemost. (2017) 15:2165–75. 10.1111/jth.13810 28834238

[17] Khorana AA Noble S Lee AYY Soff G Meyer G O'Connell C. Role of direct oral anticoagulants in the treatment of cancer-associated venous thromboembolism: guidance from the SSC of the ISTH. J Thromb Haemost. (2018) 16:1891–4. 10.1111/jth.14219 30027649

[18] Kearon C Akl EA Comerota AJ Prandoni P Bounameaux H Goldhaber SZ. Antithrombotic therapy for VTE disease: antithrombotic therapy and prevention of thrombosis, 9th ed: American College of Chest Physicians evidence-based clinical practice guidelines. Chest. (2012) 141:e419S–e96S. 10.1378/chest.11-2301 22315268 PMC3278049

[19] Stekhoven DJ Bühlmann P. Missforest-non-parametric missing value imputation for mixed-type data. Bioinformatics. (2012) 28:112–8. 10.1093/bioinformatics/btr597 22039212

[20] Vandenbroucke JP. A shortcut method for calculating the 95 per cent confidence interval of the standardized mortality ratio. Am J Epidemiol. (1982) 115:303–4. 10.1093/oxfordjournals.aje.a113306

[21] R Core Team. R: A Language and Environment for Statistical Computing. (2019). Available online at: https://www.r-project.org/ (accessed April 30, 2020).

[22] Søgaard KK Schmidt M Pedersen L Horváth-Puhó E Sørensen HT. 30-year mortality after venous thromboembolism. Circulation. (2014) 130:829–36. 10.1161/CIRCULATIONAHA.114.009107 24970783

[23] Reitter S Laczkovics C Waldhoer T Mayerhofer M Vutuc C Pabinger I. Long-term survival after venous thromboembolism: a retrospective selected cohort study among young women. Haematologica. (2010) 95:1425–8. 10.3324/haematol.2009.017616 20511663 PMC2913094

