# Supplementary material for: Long-Term Survival After Venous Thromboembolism: A Prospective Cohort Study
Source: Front Cardiovasc Med. 2021 Oct 1;8:749342. doi: 10.3389/fcvm.2021.749342 (PMC8517253; doi:10.3389/fcvm.2021.749342)
Supplement: Supplementary file 1 [file Data_Sheet_1.docx]

Supplementary Material


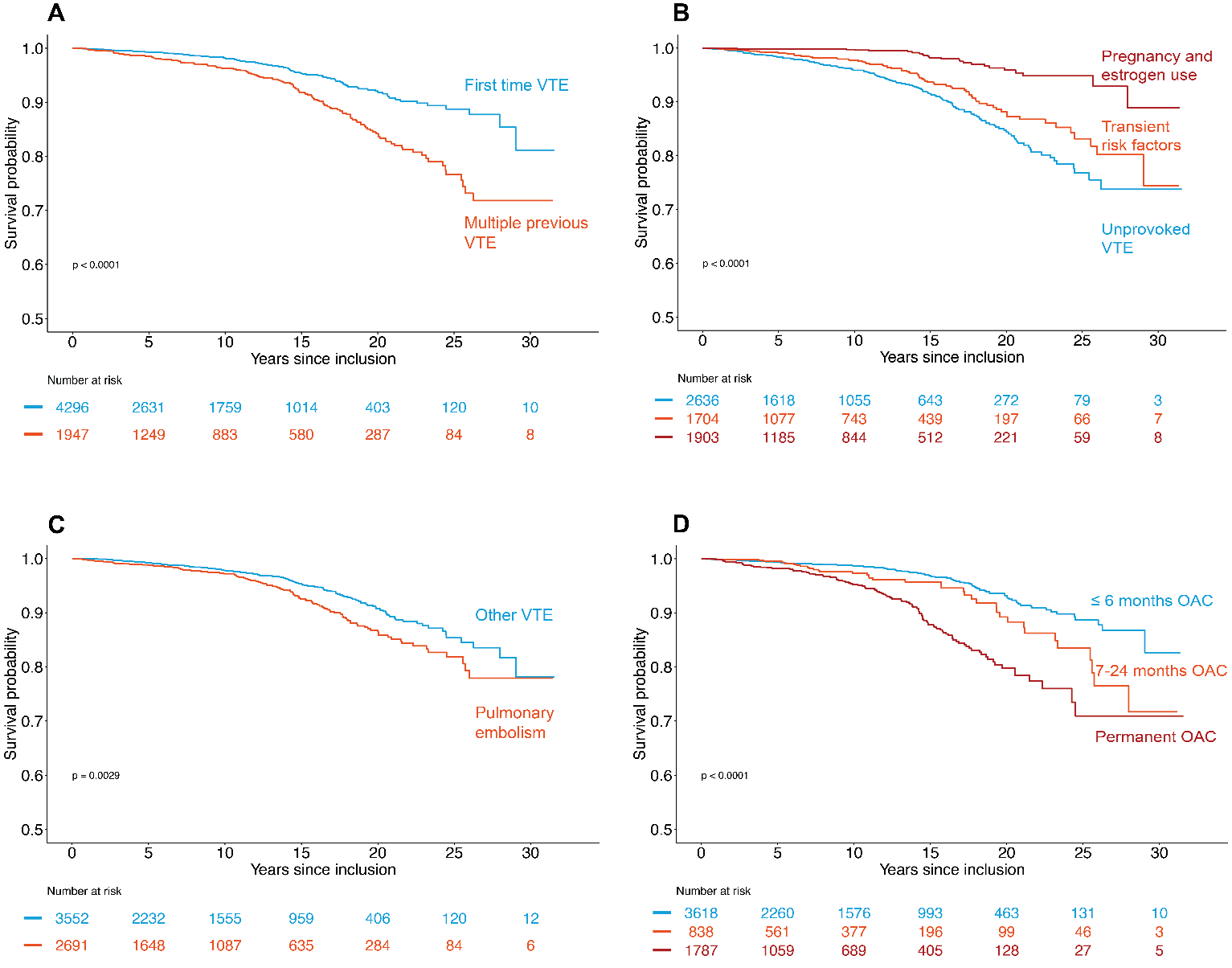


**Figure S1**: Kaplan-Meier estimates for key predictor variables relating to disease severity ([A] presence of multiple venous thromboembolic events; [B] triggering risk factors; [C] presence of pulmonary embolism), and treatment intensity ([D] duration of anticoagulation).

# Assumptions

Predictor pessimistic HR 95% CI optimistic HR 95% CI

Age 1.00 0.99, 1.00 1.08 1.07, 1.09

Sex 0.94 0.86, 1.02 1.93 1.51, 2.47

Table S1: Hazard ratio for age and sex, for a pessimistic and optimistic model.


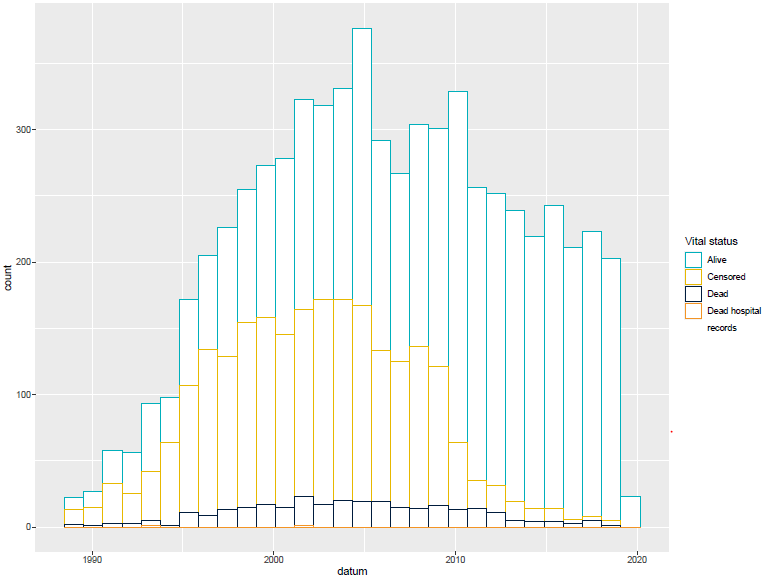


**Figure S2**: Number of patients per vital status.

Original analysis Sensitivity analysis

HR 95% CI HR 95%CI

|  |
| --- |

Age 1.09 1.08, 1.10 1.09 1.08, 1.11

Male sex 1.85 1.45, 2.38 1.31 1.02, 1.68

Index event

Deep vein thrombosis - - - -

Pulmonary embolism 1.43 1.09, 1.87 1.16 0.88, 1.52

Other VTE 0.97 0.66, 1.43 0.93 0.62, 1.38

Multiple VTE 2.07 1.61, 2.64 1.38 1.07, 1.77

Family history 0.86 0.65, 1.13 0.83 0.63, 1.11

Triggering risk factors

Pregnancy and estrogen use - - - -

Transient risk factors 3.05 2.01, 4.63 1.60 0.98, 2.62

Unprovoked VTE 4.63 3.15, 6.81 1.65 1.03, 2.66

Duration of anticoagulation therapy

| ≤ 6 months - | | - | - | - |
| --- | --- | --- | --- | --- |
| 7-24 months 1.74 | | 1.22, 2.48 | 1.49 | 1.02, 2.19 |
| permanent 3.27 | | 2.54, 4.19 | 1.69 | 1.27, 2.24 |
| Arterial thrombosis | 1.92 | 1.20, 3.06 | 0.76 | 0.39, 1.45 |
| Smoking | 0.98 | 0.74, 1.29 | 1.96 | 1.45, 2.64 |
| Obesity | 1.43 | 1.11, 1.84 | 1.15 | 0.88, 1.50 |
| Systemic disease | 1.38 | 0.92, 2.09 | 1.06 | 0.69, 1.63 |
| Diabetes mellitus | 3.97 | 2.54, 6.22 | 2.27 | 1.42, 3.62 |
| Coronary artery disease | 3.78 | 2.57, 5.58 | 1.50 | 0.99, 2.27 |
| Pulmonary disease | 1.92 | 1.19, 3.10 | 1.52 | 0.93, 2.48 |
| Hypertension | 2.93 | 2.24, 3.85 | 1.18 | 0.88, 1.58 |
| Kidney disease | 5.43 | 3.59, 8.22 | 2.48 | 1.57, 3.90 |
| Stroke | 2.58 | 1.58, 4.23 | 1.40 | 0.70, 2.79 |
| Anemia | 2.20 | 1.36, 3.56 | 2.33 | 1.41, 3.85 |

**Table S2**: Hazard ratios of the original model and a sensitivity analysis after removing all

| Predictor *x*^2^ Degrees of freedom | | | P-value |
| --- | --- | --- | --- |
| Overall | 20.55 | 21 | 0.49 |
| Age | 0.22 | 1 | 0.63 |
| Sex | 2.41 | 1 | 0.12 |
| Index event | 0.86 | 2 | 0.65 |
| Multiple VTE | 0.57 | 1 | 0.45 |
| Family history | 0.44 | 1 | 0.51 |
| Triggering risk factors | 2.59 | 2 | 0.27 |
| Duration of anticoagulation treatment | 2.04 | 2 | 0.36 |
| Arterial thrombosis | 0.44 | 1 | 0.51 |
| Smoking | 2.42 | 1 | 0.12 |
| Obesity | 3.11 | 1 | 0.08 |
| Systemic disease | 0.01 | 1 | 0.92 |
| Diabetes mellitus | 0.32 | 1 | 0.57 |
| Coronary artery disease | 0.36 | 1 | 0.55 |
| Pulmonary disease | 0.07 | 1 | 0.80 |
| Hypertension | 0.10 | 1 | 0.75 |
| Kidney disease | 0.39 | 1 | 0.53 |
| Stroke | 0.01 | 1 | 0.92 |
| Anemia | 1.45 | 1 | 0.22 |

Table S3: *x*^2^-test results of the coefficients of correlation between the Schoenfeld residuals and the transformed survival times.


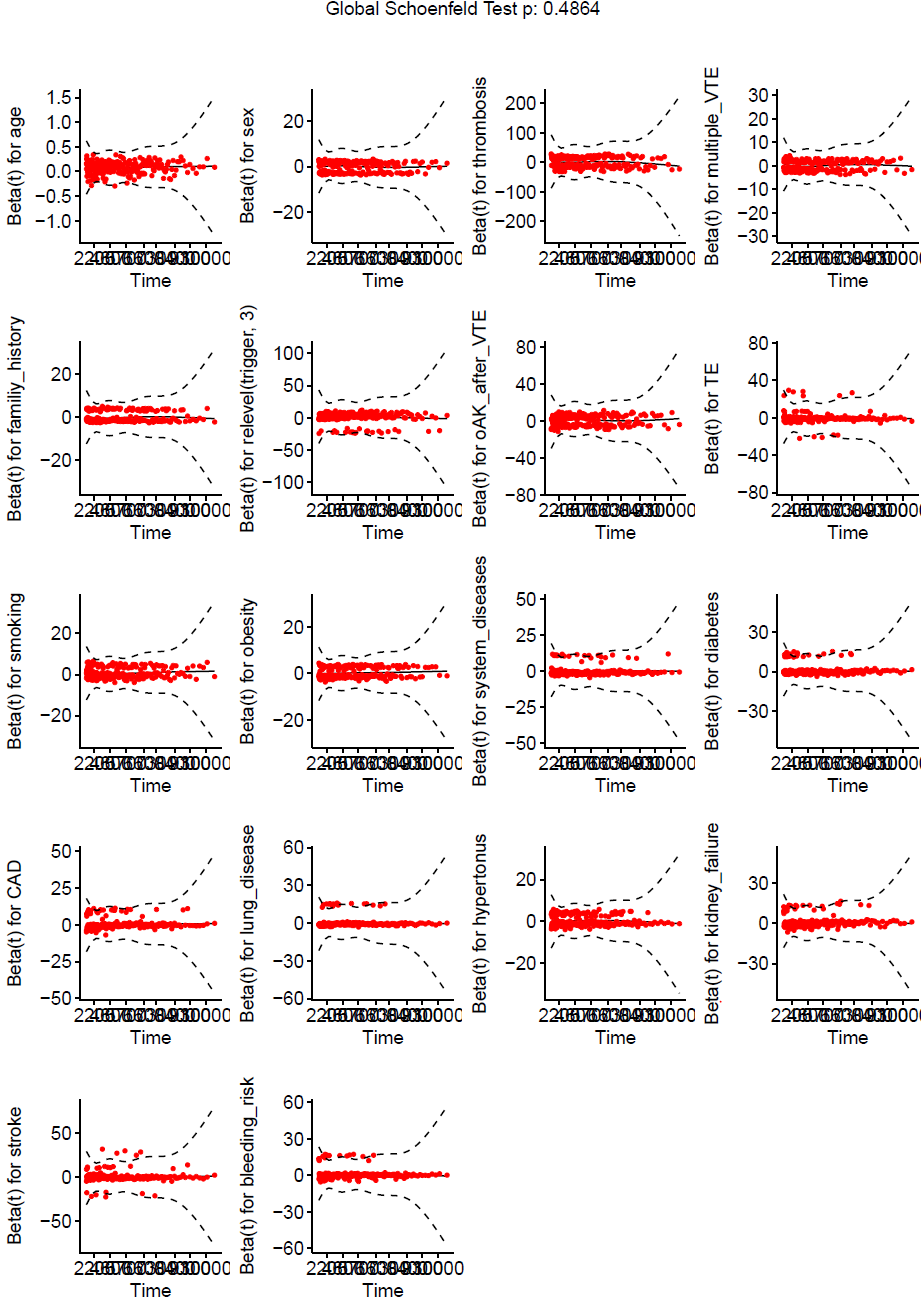


**Figure S3**: Graphs of the scaled Schoenfeld-residuals for each predictor.

|  | Full analysis | 1988 – 1997  (n = 936) | 1998 – 2007  (n = 2716) | 2008-2018  (n = 2591) |
| --- | --- | --- | --- | --- |
| Age | 1.09  (1.08, 1.11) | 1.11  (1.08, 1.15) | 1.07  (1.06, 1.09) | 1.10  (1.08, 1.13) |
| Male Sex | 1.29  (1.00, 1.65) | 0.73  (0.39, 1.38) | 1.56  (1.12, 2.19) | 1.12  (0.67, 1.85) |
| Index event |  |  |  |  |
| Deep vein thrombosis | -  - | -  - | -  - | -  - |
| Pulmonary embolism | 1.18  (0.90, 1.56) | 0.90  (0.44, 1.84) | 1.24  (0.88, 1.76) | 1.29  (0.60, 2.78) |
| Other VTE | 0.91  (0.62, 1.35) | 1.21  (0.46, 1.84) | 0.67  (0.37, 1.21) | 1.15  (0.65, 2.03) |
| Previous VTE | 1.33  (1.04, 1.71) | 1.49  (0.77, 2.88) | 1.33  (1.04, 1.71) | 1.50  (0.91, 2.47) |
| Family history | 0.87  (0.66, 1.16) | 0.94  (0.46, 1.93) | 0.96  (0.66, 1.40) | 0.64  (0.36, 1.15) |
| Triggering risk factors |  |  |  |  |
| Pregnancy and estrogen use | -  - | -  - | -  - | -  - |
| Transient risk factors | 1.63  (0.99, 2.66) | 0.59  (0.19, 1.89) | 2.12  (1.15, 3.91) | 2.61  (0.56, 12.10) |
| Unprovoked VTE | 1.69  (1.05, 2.72) | 0.90  (0.30, 2.73) | 1.78  (0.98, 3.24) | 3.42  (0.77, 15.15) |
| Duration of anticoagulation treatment |  |  |  |  |
| ≤ 6 months | -  - | -  - | -  - | -  - |
| Prolonged | 1.53  (1.04, 2.24) | 2.06  (0.92, 4.64) | 1.34  (0.75, 2.39) | 1.70  (0.81, 3.57) |
| Permanent | 1.66  (1.25, 2.21) | 1.38  (0.59, 3.25) | 1.66  (1.15, 2.39) | 1.45  (0.80, 2.63) |
| Arterial thrombosis | 0.76  (0.39, 1.46) | 1.07  (0.20, 5.85) | 0.60  (0.24, 1.49) | 0.86  (0.23, 3.27) |
| Smoking | 1.91  (1.42, 2.57) | 3.36  (1.61, 7.04) | 1.69  (1.16, 2.47) | 1.81  (0.92, 3.56) |
| Obesity | 1.15  (0.88, 1.50) | 1.93  (1.02, 3.65) | 1.29  (0.90, 1.85) | 0.81  (0.46, 1.45) |
| Systemic disease | 1.07  (0.70, 1.63) | 1.40  (0.56, 3.54) | 0.93  (0.52, 1.68) | 1.34  (0.55, 3.30) |
| Diabetes mellitus | 2.09  (1.31, 3.32) | -  -, - | 1.78  (0.97, 3.24) | 3.13  (1.41, 6.93) |
| Coronary artery disease | 1.56  (1.03, 2.37) | 2.18  (0.75, 6.34) | 1.86  (1.01, 3.42) | 0.99  (0.49, 2.01) |
| Pulmonary disease | 1.38  (0.85, 2.24) | 1.66  (0.46, 6.00) | 1.41  (0.69, 2.90) | 1.09  (0.47, 2.49) |
| Hypertension | 1.19  (0.89, 1.59) | 1.58  (0.74, 3.36) | 1.26  (0.86, 1.84) | 0.94  (0.52, 1.68) |
| Kidney disease | 2.31  (1.47, 3.63) | 3.89  (0.86, 17.67) | 1.66  (0.84, 3.31) | 2.28  (1.07, 4.87) |
| Stroke | 1.38  (0.69, 2.76) | 0.71  (0.10, 5.05) | 2.25  (0.93, 5.47) | 0.82  (0.15, 4.51) |
| Anemia | 2.34  (1.42, 3.87) | -  -, - | 1.93  (0.92, 4.08) | 3.28  (1.55, 6.97) |

**Table S4**: Multivariate sensitivity analysis by enrollment time.

|  | Full analysis | DVT and PE only |
| --- | --- | --- |
| Age | 1.09 (1.08, 1.11) | 1.09 (1.08, 1.11) |
| Male Sex | 1.29 (1.00, 1.65) | 1.22 (0.93, 1.60) |
| Index event |  |  |
| Deep vein thrombosis | - | - |
| Pulmonary embolism | 1.18 (0.90, 1.56) | 1.20 (0.91, 1.58) |
| Other VTE | 0.91 (0.62, 1.35) | - |
| Previous VTE | 1.33 (1.04, 1.71) | 1.39 (1.06, 1.82) |
| Family history | 0.87 (0.66, 1.16) | 0.88 (0.65, 1.19) |
| Triggering risk factors |  |  |
| Pregnancy and estrogen use | - | - |
| Transient risk factors | 1.63 (0.99, 2.66) | 1.68 (0.99, 2.85) |
| Unprovoked VTE | 1.69 (1.05, 2.72) | 1.83 (1.09, 3.07) |
| Duration of anticoagulation treatment |  |  |
| ≤ 6 months | - | - |
| Prolonged | 1.53 (1.04, 2.24) | 1.50 (0.98, 2.28) |
| Permanent | 1.66 (1.25, 2.21) | 1.72 (1.26, 2.33) |
| Arterial thrombosis | 0.76 (0.39, 1.46) | 0.86 (0.43, 1.71) |
| Smoking | 1.91 (1.42, 2.57) | 2.12 (1.54, 2.91) |
| Obesity | 1.15 (0.88, 1.50) | 1.13 (0.85, 1.51) |
| Systemic disease | 1.07 (0.70, 1.63) | 0.94 (0.58, 1.50) |
| Diabetes mellitus | 2.09 (1.31, 3.32) | 2.45 (2.53, 3.93) |
| Coronary artery disease | 1.56 (1.03, 2.37) | 1.49 (0.96, 2.33) |
| Pulmonary disease | 1.38 (0.85, 2.24) | 1.37 (0.83, 2.28) |
| Hypertension | 1.19 (0.89, 1.59) | 1.44 (1.06, 1.97) |
| Kidney disease | 2.31 (1.47, 3.63) | 2.61 (1.61, 4.22) |
| Stroke | 1.38 (0.69, 2.76) | 1.28 (0.62, 2.64) |
| Anemia | 2.34 (1.42, 3.87) | 2.06 (1.17, 3.63) |

**Table S5**: Sensitivity analysis using patients with PE and DVT only.
